# Telomerase activity in melanoma and non-melanoma skin cancer

**DOI:** 10.1038/sj.bjc.6690010

**Published:** 1999-09-24

**Authors:** C N Parris, S Jezzard, A Silver, R MacKie, J M McGregor, R F Newbold

**Affiliations:** Department of Biology and Biochemistry, Brunel University, Uxbridge, Middlesex, UB8 3PH, UK; National Radiological Protection Board, Chilton, Didcot, Oxon, UK; Department of Dermatology, University of Glasgow, Robertson Building, Glasgow, G12 8QQ, UK; Department of Photobiology, St John's Institute of Dermatology, St. Thomas's Hospital, London, SE1 7EH, UK

**Keywords:** skin cancer, telomerase, telomeric repeat amplification procedure, telomere length

## Abstract

Telomeres are specialized structures consisting of repeat arrays of TTAGGG*_n_* located at the ends of chromosomes. They are essential for chromosome stability and, in the majority of normal somatic cells, telomeres shorten with each cell division. Most immortalized cell lines and tumours reactivate telomerase to stabilize the shortening chromosomes. Telomerase activation is regarded as a central step in carcinogenesis and, here, we demonstrate telomerase activation in premalignant skin lesions and also in all forms of skin cancer. Telomerase activation in normal skin was a rare event, and among 16 samples of normal skin (one with a history of chronic sun exposure) 12.5% (2 out of 16) exhibited telomerase activity. One out of 16 (6.25%) benign proliferative lesions, including viral and seborrhoeic wart samples, had telomerase activity. In premalignant actinic keratoses and Bowen's disease, 42% (11 out of 26) of samples exhibited telomerase activity. In the basal cell carcinoma and cutaneous malignant melanoma (CMM) lesions, telomerase was activated in 77% (10 out of 13) and 69% (22 out of 32) respectively. However, only 25% (3 out of 12) of squamous cell carcinomas (SCC) had telomerase activity. With the exception of one SCC sample, telomerase activity in a positive control cell line derived from a fibrosarcoma (HT1080) was not inhibited when mixed with the telomerase-negative SCC or CMM extracts, indicating that, overall, *Taq* polymerase and telomerase inhibitors were not responsible for the negative results. Mean telomere hybridizing restriction fragment (TRF) analysis was performed in a number of telomerase-positive and -negative samples and, although a broad range of TRF sizes ranging from 3.6 to 17 kb was observed, a relationship between telomerase status and TRF size was not found. © 1999 Cancer Research Campaign


*British Journal of Cancer* (1999) **79**(1), 47–53
© 1999 Cancer Research Campaign

### Telomerase activity in melanoma and non-melanoma skin cancer

**CN Parris[1], S Jezzard[1], A Silver[2], R MacKie[3], JM McGregor[4] and RF Newbold[1]**

[1]Department of Biology and Biochemistry, Brunel University, Cleveland Road, Uxbridge, Middlesex UB8 3PH, UK; [2]National Radiological Protection Board, Chilton, Didcot, Oxon, UK; [3]Department of Dermatology, Robertson Building, University of Glasgow, Glasgow G12 8QQ, UK; [4]Department of Photobiology, St John's Institute of Dermatology, St. Thomas's Hospital, London SE1 7EH, UK

**Summary** Telomeres are specialized structures consisting of repeat arrays of $TTAGGG_n$ located at the ends of chromosomes. They are essential for chromosome stability and, in the majority of normal somatic cells, telomeres shorten with each cell division. Most immortalized cell lines and tumours reactivate telomerase to stabilize the shortening chromosomes. Telomerase activation is regarded as a central step in carcinogenesis and, here, we demonstrate telomerase activation in premalignant skin lesions and also in all forms of skin cancer. Telomerase activation in normal skin was a rare event, and among 16 samples of normal skin (one with a history of chronic sun exposure) 12.5% (2 out of 16) exhibited telomerase activity. One out of 16 (6.25%) benign proliferative lesions, including viral and seborrhoeic wart samples, had telomerase activity. In premalignant actinic keratoses and Bowen's disease, 42% (11 out of 26) of samples exhibited telomerase activity. In the basal cell carcinoma and cutaneous malignant melanoma (CMM) lesions, telomerase was activated in 77% (10 out of 13) and 69% (22 out of 32) respectively. However, only 25% (3 out of 12) of squamous cell carcinomas (SCC) had telomerase activity. With the exception of one SCC sample, telomerase activity in a positive control cell line derived from a fibrosarcoma (HT1080) was not inhibited when mixed with the telomerase-negative SCC or CMM extracts, indicating that, overall, *Taq* polymerase and telomerase inhibitors were not responsible for the negative results. Mean telomere hybridizing restriction fragment (TRF) analysis was performed in a number of telomerase-positive and -negative samples and, although a broad range of TRF sizes ranging from 3.6 to 17 kb was observed, a relationship between telomerase status and TRF size was not found.

**Keywords:** skin cancer; telomerase; telomeric repeat amplification procedure; telomere length

Telomeres are specialized structures located at the ends of eukaryotic chromosomes that help to stabilize chromosomes during replication (Muller, 1938; McClintock, 1941; Greider, 1994). In humans, these regions are predominantly composed of the sequence TTAGGG iterated many times to give a total of about 5–15 kbp (Moyzis et al, 1988; Morin, 1989). In normal human cells, DNA polymerase is unable to replicate the ends of linear DNA, and each cell division results in the loss of some 50–200 nucleotide from the ends of chromosomes (Allsop et al, 1995). During the lifespan of a normal somatic cell, successive cell division will result in gradual shortening of the telomeres until the chromosomes become critically short. This can lead to destabilization and loss of chromosomes followed by senescence and cell death. This telomere shortening has been suggested to be a 'mitotic clock' and a cellular mechanism whereby cells count their divisions (Harley, 1991).

The stabilization of telomere length is thought to be a critical molecular event in the multistep pathway to cellular transformation and immortalization (Rhyu, 1995). Telomerase, a ribonucleoprotein, is a key enzyme involved in the stabilization of telomeres in transformed cells in culture as well as tumour cells in vivo. Telomerase is capable of adding telomeric sequences (TTAGGG hexameric repeats) to the ends of chromosomes and, thereby, halting the erosion of chromosome with each cell division (Morin, 1989). Using the highly sensitive telomeric repeat amplification procedure (TRAP) (Piatyszek et al, 1995), telomerase activity has been detected in a large majority of immortalized cells and those derived from tumour biopsies (Kim et al, 1994; Bacchetti and Counter, 1995; Holt et al, 1996). In addition, telomerase activity has been detected in somatic cells with renewal capacity, such as germline cells of the testis and ovary, endometrial cells (Kyo et al, 1997), proliferative basal cells of the epidermis (Harle-Bachor and Boukamp, 1996) and haemopoietic cells (Broccoli et al, 1995).

Skin cancer is the most common cancer in humans, and there is increasing evidence that solar ultraviolet radiation is a major aetiological factor (IARC, 1992). The precise molecular events in skin carcinogenesis are numerous and complicated. However, inactivation of the p53 tumour-suppressor gene (TSG) is common in non-melanoma skin cancer (Brash et al, 1991), and inactivation of the p16 TSG is thought to be a critical step in melanoma development (Flores et al, 1996). In addition, evidence suggests that activation of the telomerase enzyme is a pivotal step in the development of all forms of skin cancer. In fact, telomerase activity has been observed in 75–85% of all forms of skin cancer and has also been observed in normal skin exposed to solar ultraviolet radiation (Taylor et al, 1996; Ueda et al, 1997). Thus, it is evident that stabilization and maintenance of telomeres is a central event in cellular immortalization and carcinogenesis. Moreover, evidence strongly suggests that telomerase activation is largely responsible for telomere stability (Rhyu, 1995).

However, it has been consistently demonstrated that, overall, some 15–20% of all tumour biopsy samples, including skin

*Received 19 December 1997*
*Revised 11 June 1998*
*Accepted 16 June 1998*

*Correspondence to:* CN Parris cancer, and a significant number of immortalized cell lines in culture do not have detectable telomerase activity. Therefore, in a subpopulation of cancers at least, acquisition of presumably unlimited growth potential is achieved without activation of telomerase. This critical observation suggests that other mechanisms of telomere maintenance exist, given that telomere maintenance is essential for cellular immortalization and cancer.

Without doubt, the telomerase enzyme is a central mechanism of telomere maintenance, however, pathways have been identified that appear to be independent of telomerase. The *Saccharomyces cerevisiae* Est1 mutant is able to maintain constant telomere structure by a recombination pathway using subtelomeric elements (Lundblad and Blackburn, 1993). In addition, approximately one-third of in vitro immortalized cell lines have no detectable telomerase activity, but have very long telomeres (Bryan et al, 1995). This suggests that the telomeres can be maintained by a telomerase-independent pathway, a mechanism referred to as the ALT pathway (alternative lengthening of telomeres) (Murnane et al, 1994; Bryan et al, 1997*a*). ALT has been detected in a number of human cell lines derived from tumours, in a small number of tumour biopsy samples (Bryan et al, 1997*b*), and also in cells transformed with oncogenic viruses (Bryan et al, 1995). The precise molecular pathway of the ALT mechanism is hitherto unknown, but it does not appear to involve (at least transient) telomerase activity. Telomerase-negative cell lines have been observed increasing the length of their telomeres without prior telomerase activity (Rogan et al, 1995). Moreover, the ALT mechanisms do not involve the transposition of other elements such as transposons to the telomeres, as seen in *Drosophila* because the telomeres in these cells hybridize strongly with $TTAGGG_n$ probes (Bryan et al, 1995).

Furthermore, it has been recently demonstrated in transgenic mice, which are deleted for the RNA component of the telomerase enzyme (*mTR*), that although telomerase was central for telomere maintenance it was not required for establishment of continuous cell lines, oncogenic transformation or tumour formation in mice (Blasco et al, 1997).

Therefore, it is clear that telomere maintenance in some immortal cell lines and tumour samples can occur by molecular mechanisms that do not involve the telomerase enzyme, although the precise events have yet to be described.

In this study, we demonstrate telomerase activity in premalignant lesions and skin exposed to solar UV irradiation. We also present evidence implicating the activation of telomerase in both melanoma and non-melanoma tumours of the skin. In addition, a collection of squamous cell carcinomas with no detectable telomerase activity has been identified. The telomere length of some telomerase-positive and -negative skin cancer samples was examined to determine whether there is a relationship between telomere length and telomerase activity.

### MATERIALS AND METHODS

### Tissue samples

Thirty-two cutaneous malignant melanoma tumour samples were obtained from the Department of Dermatology, University of Glasgow, UK. All samples with the exception of one primary lesion, were derived from lymph node metastases (clinical stage 3 disease). In addition, the single primary tumour was accompanied by two metastatic lesions from the same individual. The remaining 83 tissue samples were obtained by 6-mm punch biopsy from St. John's Institute of Dermatology, London, UK. The non-melanoma squamous cell carcinoma (SCC) and basal cell carcinoma (BCC) samples were of moderate to well-differentiated nodular lesions. The collected tissues were subject to histopathological examination to verify the diagnosis in each case. Tissues were snap frozen in liquid nitrogen and stored at –80°C until use. The samples are listed in Table 1.

### Extract preparation

Samples were removed from –80°C storage, and after detailed visual inspection, were carefully trimmed to remove excess normal tissue. A longitudinal section of the tissue sample was first prepared, thus ensuring that all cell types within the sample were included in the extract preparation. The section was then carefully sliced on 100-cm Petri dishes using sterile disposable scalpels and homogenized with about 10–12 strokes in a Dounce homogenizer containing ice-cold lysis buffer [10 mM tris-HCl, pH 7.5, 1 mM magnesium chloride, 1 mM EGTA, 0.5% (w/v) CHAPS, 10% (v/v) glycerol, 0.1 mM PMSF and 5 mM β-mercaptoethanol]. After a further 30 min incubation on ice, lysates were transferred to 1.5-ml centrifuge tubes and spun at 14 000 ***g*** for 30 min at 4°C. The supernatants were snap frozen and stored at –80°C. Protein concentrations were determined using the Coomassie protein kit (Pierce) and aliquots were prepared at a concentration of 0.5 μg $\mu l^{-1}$ protein.

### Telomerase assay

Telomerase activity was determined using a two-stage assay modified from that of Kim et al. (1994) Assay tubes consisted of 1 μl CX primer (0.1 μg) (5′-CCCTTACCCTTACCCTTACCCTAA-3′) lyophilized under a wax barrier (Ampliwax PCR gem 100, Perkin-Elmer), above which a reaction mixture containing 20 mM tris-HCl, pH 8.3, 1.5 mM magnesium chloride, 68 mM potassium chloride, 0.05% Tween-20, 1 mM EGTA, 50 mM dNTPs, 0.1 μg of TS primer (5′-AATCCGTCGAGCAGAGTT-3′), 0.2 μCi dCTP and 0.2 μCi TTP (3000 Ci $mmol^{-1}$) (ICN Flow), 0.5 μg T4g32 single-stranded binding protein (Pharmacia), 2 U *Taq* Polymerase (Promega) and 1 μg of extract was added. Included in each assay was 5 ag of an internal telomerase amplification standard (ITAS) (a gift from L Gollahon) to detect false-negative tumour samples containing *Taq* inhibitors. Extension of the TS primer by

**Table 1** Summary of telomerase activity in skin biopsy samples

| Sample type | Number | Positive |
|---|---|---|
| Normal skin | 16 | 2 |
| Benign proliferative lesions | | |
| Viral/seborrhoeic wart | 16 | 1 |
| Premalignant SCC lesions | | |
| Bowen's disease | 4 | 1 |
| Actinic keratoses | 22 | 10 |
| Non-CMM and CMM lesions | | |
| SCC | 12 | 3 |
| BCC | 13 | 10 |
| CMM | 32 | 22 |
| Total | 115 | 49 |

 © Cancer Research Campaign 1999

telomerase was conducted at room temperature and the reaction mixture was heated to 90°C for 90 s. Polymerase chain reaction (PCR) conditions were as follows: 94°C for 30 s, 50°C for 40 s and 72°C for 50 s for 31 cycles. Telomerase products were separated on 10% non-denaturing polyacrylamide gels and visualized using a PhosphoImager (Molecular Dynamics).

### Alkaline phosphatase estimation

The viability of the protein components of extracted biopsy samples was estimated by determining the activity of alkaline phosphatase as previously described (Piatyszek et al, 1995). In brief, 10-μl aliquots of protein extract in TRAP lysis buffer containing 20–30 μg protein was added to 200 μl *p*-nitrophenyl phosphate substrate solution and incubated for 30 min at 37°C. The reaction was stopped with 2 ml 0.005 N sodium hydroxide, after which the absorbance at 405 nm was read. Concentrated hydrochloric acid (40 μl) was added to remove the colour due to *p*-nitrophenol, after which the residual absorbance at 405 nm was subtracted to give absorbance attributable to alkaline phosphatase.

### Telomerase inhibitors

To exclude the possibility of false-negative TRAP assay results, we performed mixing experiments to test for the presence of telomerase inhibitors introduced during extract preparation. Extracts derived from skin sample biopsies, with or without telomerase activity, were mixed in a 1:1 ratio with extracts prepared from a telomerase-positive fibrosarcoma cell line HT1080. The mixtures were subjected to TRAP analysis as described previously.

### TRAP sensitivity experiments

The sensitivity of our TRAP assay was routinely determined by using the telomerase-positive HT1080 cell extract. Dilutions from 1 to 1000 cell equivalents of extract were made, and at each dilution telomerase activity was determined with the TRAP assay. TRAP assay sensitivity was deemed sufficient if telomerase activity in ten cell equivalents was detected.

### Telomere length analysis

Tumour biopsy samples were examined macroscopically and trimmed of excess tissue to leave an homogeneous sample of tumour tissue. High molecular weight DNA was then prepared by isolating the nuclei. Briefly, nuclei were isolated by 10–12 strokes of Dounce homogenization using a loose-fitting type B pestle in ten volumes of ice-cold nuclear extraction buffer [250 mM sucrose, 10 mM tris HCl, pH 8, 10 mM magnesium chloride, 1 mM EGTA, 10 mg $ml^{-1}$ bovine serum albumin (BSA), 0.2 mM PMSF, 1 mM DTT]. Supernatants were recovered after centrifugation at 900 r.p.m. for 5 min and 1:7 vol of 80% glycerol in nuclear extraction buffer was added. Nuclei were pelleted at 3500 r.p.m. for 15 min and washed with a further 15 ml of nuclear extraction buffer. Nuclei were counted and approximately $1\times10^6$ were embedded in 1% low gelling temperature agarose (Flowgen). Agarose plugs were then processed according to Anand (1986) and the DNA in plugs digested with 80 U *Hin*f1 for 4 h at 37°C, as described previously (Silver et al, 1991).

DNA was fractionated though a 1% agarose gel in 0.5 × Tris-borate-EDTA buffer by pulsed field gel electrophoresis (PFGE) using a CHEF mapper system (Biorad). The conditions for the PFGE were set to allow resolution of DNA fragments ranging from 2 to 50 kb using the autoalgorithm according to the manufacturer's instructions. DNA standard markers were obtained from BRL, Life Technologies. DNA was visualized by ethidium bromide staining and transferred to Hybond $N^+$ membranes (Amersham) overnight using 0.4 N sodium hydroxide. Membranes were hybridized overnight to a $\gamma$-$^{32}$P 5′ end-labelled telomeric probe [$\gamma$-$^{32}$P-$(TTAGGG)_4$] at 48°C. Membranes were washed four times in 4×SSC, 0.1% sodium dodecyl sulphate (SDS), at 48°C for 15 min and once in 2×SSC, 0.1% SDS, at room temperature for 30 min. Hybridized membranes were then autoradiographed overnight using intensifying screens. Mean telomere lengths of the samples were estimated by densitometric scanning of autoradiographs using a Biorad GS700 imaging densitometer and then calculated as detailed in Hastie et al (1990).

Mean telomere lengths were determined as described by Hastie et al. (1990). Briefly, autoradiographs were analysed by an automatic autoradiographic scanner (Biorad). The optical density reading under the peak was integrated and the mean determined.

### RESULTS

### Telomerase activity

#### *Normal skin and benign cutaneous lesions*

Using the TRAP protocol, telomerase activity was determined in a total of 115 skin samples, comprising normal skin, cutaneous viral warts, seborrhoeic keratoses, premalignant keratoses, squamous and basal cell carcinomas and cutaneous malignant melanomas. The samples and corresponding telomerase status are summarized in Table 1. The majority (14 out of 16) of normal skin samples were negative for telomerase activity. One of the normal skin samples, which proved telomerase positive, was from chronically sun-exposed skin, consistent with previous findings demonstrating transient telomerase activation associated with solar UV exposure (Taylor et al, 1996; Ueda et al, 1997).

A total of 16 samples derived from viral and seborrhoeic warts were analysed for telomerase activity, and only one (6.25%) of the samples was positive. The other 15 samples had no detectable telomerase activity.

#### *Premalignant lesions*

Premalignant lesions, including Bowen's disease and actinic keratoses, were examined to test whether telomerase was activated at this stage in skin carcinogenesis. Ten out of 22 samples (45%) assayed for telomerase activity were positive. The data indicated that telomerase activity was detectable in a variety of non-malignant proliferative disorders and, moreover, that telomerase was also activated in substantial numbers of premalignant lesions of squamous cell carcinoma of the skin.

#### *Invasive melanoma and non-melanoma skin cancers*

Thirteen BCC, 12 SCC and 32 cutaneous malignant melanoma (CMM) samples were analysed for the presence of telomerase activity. The majority of BCCs (10 out of 13; 77%) showed telomerase activity, results consistent with previous observations (Taylor et al, 1996; Ueda et al, 1997), but only three SCC samples had detectable telomerase activity (3 out of 12; 25%). This result is in contrast to the previous studies of Taylor et al (1996) and Ueda et al (1997), who found telomerase activity in 75–85% of SCCs.

© Cancer Research Campaign 1999 

To exclude the possibility of a lack of assay sensitivity at low protein concentrations, repeat TRAP assays were performed at several different protein extract dilutions (0.25, 0.5, 1.0 and 2.0 μg $ml^{-1}$). However, at all concentrations of extract, 9 out of 12 (75%) of the SCC samples proved to be telomerase negative.

In contrast, telomerase activation proved to be a more consistent event in CMM, with 69% (22 out of 32) demonstrating enzyme activity. However, a significant number (10 out of 32) still did not give positive TRAP results. Indeed, of the negative CMM samples, three biopsies were obtained from the same individual and included a primary and two independent metastatic lesions biopsied from distant sites and all failed to show telomerase activation. This result clearly demonstrates that in some cases of advanced CMM, including metastatic lesions, there is a failure to activate the telomerase enzyme.

### Telomerase inhibition experiments

In view of the significant number of telomerase-negative SCC, CMM and, to a lesser extent, BCC samples, it was possible that telomerase or *Taq* polymerase inhibitors were present in the protein extracts. Such inhibitors would obviously create inaccurate false-negative results. To eliminate this possibility, negative extracts were mixed with a telomerase-positive control protein extract derived from the HT1080 fibrosarcoma cell line. As a consequence, inhibitors of the reaction would be identified by the disappearance or reduction in intensity of the telomerase ladder and/or ITAS band after PCR and electrophoresis.

Telomerase-negative SCC, CMM and BCC protein extracts were mixed in a 1:1 ratio with the telomerase-positive HT1080 cell extract. In addition, mixing of HT1080 extract with telomerase-negative normal skin and BSA was also performed. Of the seven SCC samples used in the mixing experiments, only one appeared to have a *Taq* polymerase inhibitor present, as demonstrated by the lack of an ITAS band and no telomerase ladder (Figure 1A, lane 2). None of the telomerase-negative normal skin samples contained either a *Taq* or telomerase inhibitor (Figure 1B, lanes 9 and 10) and, thus, were bona fide telomerase-negative samples. Moreover, the addition of a non-specific protein (BSA) to the HT1080 extract failed to inhibit or to reduce telomerase activity (Figure 1, lane 11).

Telomerase/*Taq* inhibition experiments were also performed with extracts from three telomerase-negative CMM samples, in which telomerase activity was not detected (Figure 1B). Similar to the SCC samples, the CMMs contained neither a telomerase or *Taq* polymerase inhibitor and, hence, were taken to be genuine telomerase-negative CMM tumours.

In summary, the lack of telomerase activity in the SCC, CMM and BCC cancer samples was not due to inhibitors present in the protein samples. Finally, the alkaline phosphatase activity of the telomerase-negative samples was not different from protein extracts derived from equivalent telomerase-positive samples.

### Sensitivity of the TRAP assay – dilution experiments

To exclude the possibility that an overall lack of TRAP assay sensitivity was responsible for negative results, dilution experiments were routinely performed to determine the minimum number of cell equivalents required to give a positive TRAP signal. Using the HT1080 fibrosarcoma cell line, we were able to routinely detect telomerase activity in a minimum of ten cell equivalents (Figure 2, lane 3). However, maximum telomerase activity was normally observed in 250–750 cell equivalents (Figure 2, lanes 6–8). In view of the finding that we were able to detect telomerase activity in as little as ten cell equivalents, we were satisfied that our assay was of sufficient sensitivity for these experiments.

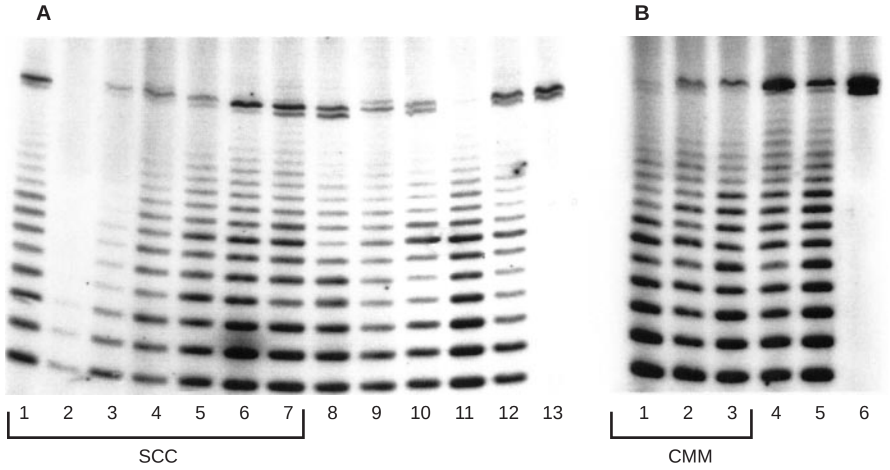

**Figure 1** (**A**) Inhibition of telomerase activity by telomerase-negative biopsy samples. Protein extracts were mixed in a 1:1 ratio with the telomerase-positive HT1080 cell extract. Lanes 1 and 3–7, telomerase-negative SCC samples which do not inhibit the telomerase activity of the positive HT1080 extract; lane 2, telomerase-negative SCC which does inhibit the positive HT1080 extract; lane 8, telomerase-negative BCC sample; lanes 9 and 10, telomerase-negative normal skin (extracts do not inhibit the HT1080 telomerase activity); lane 11, BSA; lane 12, telomerase activity of the HT1080 extract alone (positive control); lane 13, lysis buffer (negative control). (**B**) Failure of the telomerase negative extracts of CMM to inhibit the telomerase-positive HT1080 extracts in a mixing experiment. Lanes 1–3, telomerase-negative CMM extracts mixed with HT1080 extract in a 1:1 ratio; lane 4, BSA; lane 5, HT1080 extract (positive control); lane 6, lysis buffer (negative control)

 © Cancer Research Campaign 1999

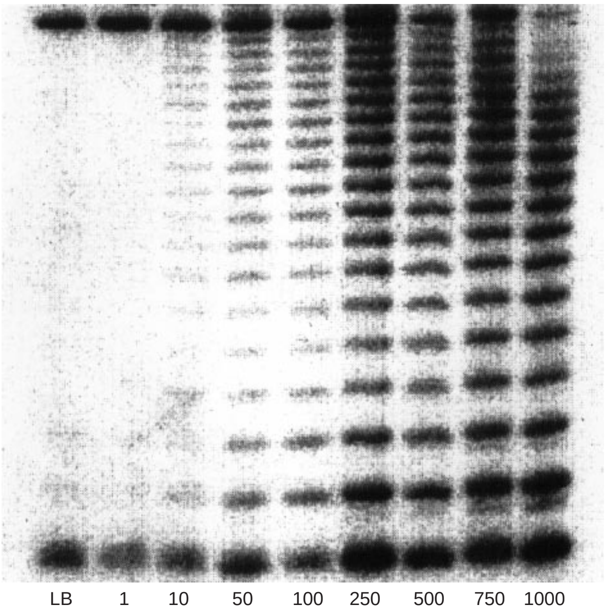

**Figure 2** Sensitivity of the TRAP assay. A representative example of the dilution experiment in which telomerase-positive HT1080 extract was diluted from 1000 to 1 cell equivalents and the corresponding telomerase activity determined. Telomerase activity could be detected in as little as ten cell equivalents of extract

### Mean telomere restriction fragment size

Telomere size was estimated in six telomerase-negative and two telomerase-positive SCC samples, two telomerase-positive CMM and a normal skin sample (Figure 3A and B and Figure 4). A wide range of telomere hybridizing restriction fragment (TRF) size was observed, suggesting that there did not appear to be a relationship between telomerase activity and TRF size for any of these samples. For the telomerase-negative SCCs, the mean TRF size ranged from 3.6 to 11 kb, and two telomerase-positive SCCs exhibited a similar size diversity with one sample of 17 kb and another of 4.1 kb. Also, the two telomerase-positive CMM samples had TRF sizes of 4.5 and 14.2 kb respectively.

### DISCUSSION

We have investigated the activity of the ribonucleoprotein telomerase in a large, random collection of different skin lesions, and examined the relationship between malignancy in this tissue and telomerase reactivation.

In normal skin, only 2 out of 16 (12.5%) had detectable telomerase activity. One example was observed in chronically sun-exposed skin, in accord with previous data, demonstrating telomerase reactivation and prior solar UV exposure (Taylor et al, 1996; Ueda et al, 1997). Although the infiltration of lymphocytes into the skin may cause transient telomerase activity in skin biopsy samples (Broccoli et al, 1995), in this study none of the normal skin samples were observed with lymphocytic infiltration by histopathological examination. However, it should be stated that telomerase activity in normal skin samples is not without precedence because telomerase activity has been demonstrated in some normal samples, which could be attributed to proliferating basal cells of the epidermis (Harle-Bachor and Boukamp, 1996).

Telomerase activity was similarly rare in 16 benign proliferative skin lesions, namely the viral and seborrhoeic wart samples (Table 1). Of the premalignant squamous cell lesions (actinic keratoses and Bowen's disease), a significant number were found to be telomerase positive, suggesting that telomerase reactivation may occur as a preinvasive event in skin carcinogenesis.

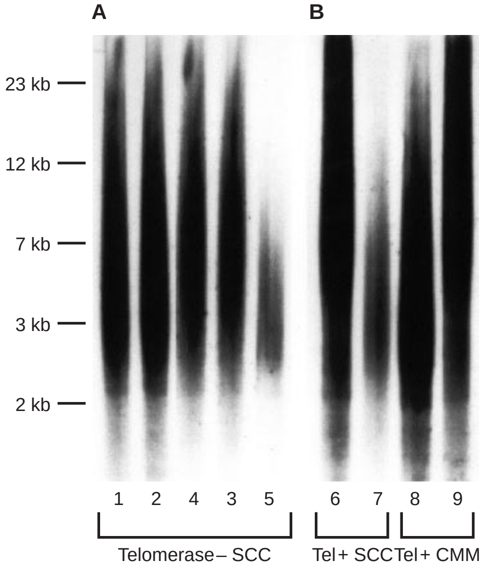

**Figure 3** Southern blot of telomere-hybridizing restriction fragments derived from telomerase-positive and -negative SCC samples and telomerase-positive CMM samples. (**A**) Lanes 1–5, telomere-hybridizing restriction fragments derived from telomerase-negative SCC samples. (**B**) Lanes 6 and 7, telomerase-positive SCC samples and lanes 8 and 9 telomerase-positive CMM samples

© Cancer Research Campaign 1999

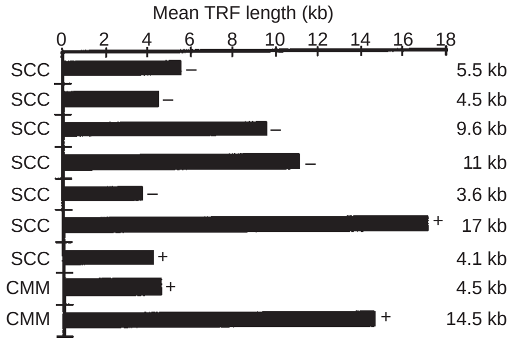

**Figure 4** Histogram of the mean telomere-hybridizing restriction fragment length derived from the data of Figure 3. Lanes 1–5 are representative of telomerase-negative SCC. Lanes 6 and 7 are representative of telomerase-positive SCC samples and lanes 8 and 9 telomerase-positive CMM samples

In the malignant skin condition SCC, telomerase activity was shown by Taylor et al (1996) and Ueda et al (1997) to be detectable in some 75–85% of samples assayed. In contrast, the data presented here showed only 25% of SCC samples with discernible telomerase activity. Considerable care was taken to ensure that our observations were not artefactual, particularly in view of Taylor et al (1996) reporting that telomerase activity in SCC is generally lower than in other histological types of tumour such as BCC. Hence, titration experiments with known positive cell extract (HT1080) was regularly performed and were shown to detect telomerase activity in as few as ten cell equivalents (Figure 2). In addition, the lack of telomerase activity could not be explained by inactive protein samples because all the protein extract samples used in this study had similar alkaline phosphatase activities. False-negative TRAP assay results can be caused by the presence of a telomerase or *Taq* polymerase inhibitor, and inhibitors may be naturally present or introduced during sample preparation. To eliminate the possibility of false-negative results, mixing experiments with a positive control (HT1080 cells) and the negative cell extracts were performed. Only one negative SCC sample was identified as a false negative, and its removal from the data represents a small change in the overall frequency of telomerase-positive SCC samples (25% $\rightarrow$ 27%). In conclusion, the high proportion of negative SCC samples were not due to experimental artefact.

In contrast to the SCCs, 69% of CMMs examined were positive for telomerase. This higher value might be explained by the greater overall tumour aggression and more frequent metastasis associated with CMM. In this respect, telomerase activity may be explained by the concept of 'mortal' tumours. In SCCs, in which there is limited aggression and a lower metastatic rate, the lesion might be composed of transformed cells that are not immortalized, thereby explaining the lack of telomerase activity in the majority of SCC tumours. However, it remains an open question whether SCCs with telomerase activity are any more aggressive than those without. Given the phenotype of CMM, it is unlikely that this would be achieved without immortalization of cells and yet, in a significant number of CMM samples, telomerase activity was not detected. For example, in three samples of CMM derived from the same individual (one primary and two metastatic), telomerase was not observed, providing strong evidence that the acquisition of tumorigenic and metastatic potential can be achieved in the absence of telomerase activity.

Given that we have identified a number of SCC and CMM samples that lack functional telomerase activity, it was appropriate to ask what effect the absence of activity had on telomere length. It was observed that telomere length could not be related to telomerase activity (Figures 3 and 4). For example, telomerase-positive samples were among the tumours with the largest and smallest mean TRF length (kb). Also, the negative SCC had mean TRFs ranging from 3.6 to 11 kb. Our data are consistent with the findings of Wainwright et al (1995), who demonstrated that telomere length varied significantly within samples derived from BCCs despite the presence of telomerase activity in these tumours.

If telomere stabilization is critical in skin carcinogenesis, it may be that mechanisms other than telomerase activation are involved in telomere maintenance. In the *S. cerevisiae* mutant Est1, telomeres are maintained via a recombination pathway using subtelomeric elements (Lundblad and Blackburn, 1993). Moreover, in the yeast *Kluyveromyces lactis*, telomeres can be maintained by a RAD52-dependent recombination mechanism during which telomeric repeat sequences are added, in contrast to subtelomeric elements as in *S. cerevisiae* (McEachern and Blackburn, 1996). *Drosophila*, however, can utilize transposable elements to maintain telomere length (Bryan et al, 1995).

An ALT (alternative lengthening of telomeres) pathway has been proposed (Murnane et al, 1994; Bryan et al, 1997) to explain the observation that approximately one-third of in vitro immortalized cell lines and an increasing number of tumour biopsy samples lack telomerase activity. The precise molecular events of the ALT pathway are not fully understood, but, in general, cells utilizing this pathway have long heterogeneous telomeres (Bryan et al, 1997). Three of our telomerase-negative SCCs do not fall into this category and have relatively short telomeres (3.6, 4.5 and 5.5 kb). In this respect, the negative SCCs do not fulfil a central criterion of the ALT pathway.

In summary, we have identified a number of SCC and CMM samples which are apparently lacking telomerase activity. Moreover, our data show there is no relationship between telomere size and telomerase activity in the malignant skin lesions analysed. These data add to an increasing number of reports in which the absence of telomerase activity has been associated with tumour biopsies (e.g. Bryan et al, 1995). This suggests that telomerase-negative tumour cells can utilize a telomerase-independent pathway to maintain telomere size. However, the precise means by which maintenance is achieved remains to be understood.

### ACKNOWLEDGEMENT

This work was supported by EC Research Contract: ENV4-CT96-0172.

### REFERENCES

Allsop RC, Chang E, Kashafi-Aazam, Rogaeu EI, Piatyszek MA, Shay JW and Harley CB (1995) Telomere shortening is associated with cell division *in vitro* and *in vivo*. *Exp Cell Res* **220**: 194–200

Anand R (1986) Pulsed field gel electrophoresis: a technique for fractionating large DNA molecules. *Trends Genet* **2**: 278–283

Bacchetti S and Counter CM (1995) Telomeres and telomerase in human cancer. *Int J Oncol* **7**: 423–432

Blasco MA, Lee H-W, Prakash-Hande M, Samper E, Landsdorp PM, DePinho RA and Greider CW (1997) Telomere shortening and tumour formation by mouse cells lacking telomerase RNA. *Cell* **91**: 25–34

Brash DE, Rudolph JA, Simon JA, Lin A, McKenna GJ, Baden HP, Halperin AJ and Ponten J (1991) A role for sunlight in skin cancer: UV-induced p53 mutations in squamous cell carcinoma. *Proc Natl Acad Sci USA* **88**: 10124–10128

 © Cancer Research Campaign 1999

Broccoli D, Young JW, and de Lange T (1995) Telomerase activity in normal and malignant hemopoietic cells. *Proc Natl Acad Sci USA* **92**: 9082–9086

Bryan TM, Englezou A, Gupta J, Bacchetti S and Reddel RR (1995) Telomere elongation in immortal human cells without detectable telomerase activity. *EMBO J* **14**: 4240–4248

Bryan TM, Marusic L, Bacchetti S, Namba M and Reddel RR (1997*a*) The telomere lengthening mechanism in telomerase-negative immortal human cells does not involve the telomerase RNA subunit. *Hum Mol Genet* **6**: 921–926

Bryan TM, Englezou A, Dalla-Pozzah L, Dunham MA and Reddel RR (1997*b*) Evidence for an alternative mechanism for maintaining telomere length in human tumors and tumor-derived cell lines. *Nature Med* **3**: 1271–1274

Flores JF, Walker GJ, Glendening JM, Halsuka FG, Castresana JS, Rubio MP, Pastorfide GC, Boyer LA, Kao WH, Bulyk ML, Barnhill RL, Hayward NK, Housmann DE and Fountain JW (1996) Loss of the $p16^{INK4a}$ and $p15^{INK4b}$ genes, as well as neighboring 9p21 markers in sporadic melanoma. *Cancer Res* **56:** 5023–5032

Greider CW (1994) Mammalian telomere dynamics: healing fragmentation, shortening and stabilization. *Curr Opin Genet Dev* **4**: 203–211

Harle-Bachor C and Boukamp P (1996) Telomerase activity in the regenerative basal layer of the epidermis in human skin and in immortal and carcinoma-derived skin keratinocytes. *Proc Natl Acad Sci USA* **93**: 6476–6481

Harley CB (1991) Telomere loss: mitotic clock or genetic time bomb. *Mutat Res* **256**: 271–282

Hastie ND, Dempster M, Dunlop MG, Thompson AM, Green DK and Allshire RC (1990) Telomere reduction in human colorectal carcinoma and with ageing. *Nature* **346**: 866–868

Holt SE, Wright WE and Shay JW (1996) Regulation of telomerase activity in immortal cell lines. *Mol Cell Biol* **16**: 2932–2939

IARC (1992) Monograph on the evaluation of carcinogenic risk to humans. In *Solar and Ultraviolet Radiation*, vol. 55. IARC; Lyon

Kim NW, Piatyszek MA, Prowse KR, Harley CB, West MD, Ho PLC, Coviello GM, Wright WE, Weinrich SL and Shay JW (1994) Specific association of human telomerase activity with immortal cells and cancer. *Science* **226**: 2011–2015

Kyo S, Takakura M, Kohama T and Inoue M (1997) Telomerase activity in human endometrium. *Cancer Res* **57**: 610–614

Lundblad V and Blackburn EH (1993) An alternative pathway for yeast telomere maintenance rescues *estl*$^{-}$ senescence. *Cell* **73:** 347–360

McClintock B (1941) The stability of broken ends of chromosomes in *Zea mays*. *Genetics* **41**: 234–282

McEachern MJ and Blackburn EH (1996) Cap-prevented recombination between terminal telomeric repeat arrays (telomere CPR) maintains telomeres in *Kluyveromyces lactis* lacking telomerase. *Genes Dev* **10**: 1822–1834

Morin GB (1989) The human telomere terminal transferase enzyme is a ribonucleoprotein that synthesizes TTAGGG repeats. *Cell* **59**: 521–529

Moyzis RK, Buckingham JM, Cram LS, Dani M, Deaven LL, Jones MD, Meyne J, Ratliff RL and Wu JR (1988) A highly conserved repetitive DNA sequence, $(TTAGGG)_n$, present at the telomeres of human chromosomes. *Proc Natl Acad Sci USA* **85**: 6622–6626

Muller HJ (1938) The remaking of chromosomes. The collecting net. *Woods Hole* **13**: 181–198

Murnane JP, Sabatier L, Marder BA and Morgan WF (1994) Telomere dynamics in an immortal human cell line. *EMBO J* **13**: 4953–4962

Piatyszek MA, Kim NW, Weinrich SL, Himaya K, Himaya E, Wright WE and Shay JW (1995) Detection of telomerase activity in human cells and tumours by a telomeric repeat amplifictaion protocol (TRAP). *Methods Cell Sci* **17**: 1–15

Rogan EM, Bryan TM, Hukku B, Maclean K, Chang AC-M, Moy EL, Englezou A, Warneford SG, Dalla-Pozza L and Reddel RR (1995) Alterations in p53 and p16INK4 expression and telomere length during spontaneous immortalisation of Li–Fraumeni syndrome fibroblasts. *Mol Cell Biol* **15**: 4745–4753

Rhyu MS (1995) Telomeres, telomerase, and immortality. *J Natl Cancer Inst* **87**: 884–894

Silver A, George A, Masson W, Breckon G, Adam J and Cox R (1991) DNA methylation changes in the IL-1 (2F) chromosomal region of radiation induced acute myeloid leukaemias carrying chromosome 2 rearrangements. *Genes Chromosomes Cancer* **3:** 376–381

Taylor RS, Ramirez RD, Ogoshi M, Chaffins M, Piatyszek MA and Shay JW (1996) Detection of telomerase activity in malignant and non-malignant skin conditions. *J Invest Dermatol* **106**: 759–765

Ueda M, Ouhtit A, Bito T, Nakazawa K, Lubbe J, Ichihashi M, Yamasaki H and Nakazawa H (1997) Evidence for UV-associated activation of telomerase in human skin. *Cancer Res* **57**: 370–374

Wainwright LJ, Middleton PG and Rees JL (1995) Changes in mean telomere length in basal cell carcinomas of the skin. *Genes Chromes Cancer* **12**: 45–49

*© Cancer Research Campaign 1999*
